# Comparison of intravascular lithotripsy versus rotational atherectomy for the treatment of severe coronary artery calcification

**DOI:** 10.1186/s12872-024-03965-1

**Published:** 2024-06-19

**Authors:** Yuhao Zhao, Ping Wang, Ze Zheng, Yuchen Shi, Jinghua Liu

**Affiliations:** Center for Coronary Artery Disease(CCAD), Beijing Anzhen Hospital, Capital Medical University, Beijing Institute of Heart, Lung and Blood Vessel Diseases, Beijing, China

**Keywords:** Severe coronary artery calcification, Percutaneous coronary intervention, Coronary intravascular lithotripsy, Rotational atherectomy, Major advent cardiovascular event

## Abstract

**Background:**

Calcified lesions are one of the most challenging cases for PCI, where optimal angiographic results and satisfying outcomes are hard to achieve.

**Methods:**

We evaluated the baseline clinical, procedures characteristics and outcomes of patients with severe coronary artery calcification (CAC) who underwent coronary intravascular lithotripsy (IVL) and rotational atherectomy (RA).

**Results:**

Respectively 152 and 238 patients who underwent IVL and RA are enrolled from January 2023 to November 2023. Regarding demographic characteristics, the gender proportion, medical history of PCI and smoke history among groups reach statistical significance. Left anterior descending and right coronary artery were the main vessels treated in both groups. The 2.5 and 3.0 mm IVL balloons and 1.5 mm burr were the most commonly used. 99.3% cases were successfully implanted drug-eluting stents after IVL balloon pre-treatment, which was higher than in the group treated with RA. During hospitalization, there were no serious adverse events in the IVL group, but there were two adverse events in the RA group. Procedural complications were higher in the RA group than the IVL group (5.5% vs. 0.7%, *P* = 0.027).

**Conclusions:**

IVL appears to be safe and effective for the treatment of severe CAC lesions compared to RA.

## Introduction

Severe coronary artery calcification (CAC) increases the complexity of percutaneous coronary intervention (PCI) which presents a unique challenge to interventional cardiologists. The incidence of CAC was detected in 38% of all patients during angiography and 74% when intravascular ultrasound (IVUS) was used [1]. Advanced aged and the increase of diabetes, hypertension and renal insufficiency is the cause of the increase of the incidence rate and severity of vascular calcification [2, 3]. The combination of PCI and drug eluting stent (DES) implantation is currently the most common mode of coronary revascularization. However, the severe CAC patients who undergo PCI have worse clinical outcomes [4]. Traditionally, high pressure dilatation of non-compliant balloons, cutting/scoring balloons and atherectomy (orbital, rotational or laser) have been used to treat severe CAC, but PCI of severe CAC have inherent limitations, such as early complications (perforation, dissection, stent rupture, myocardial infarction) or late adverse events (restenosis, stent thrombosis, and repeated revascularization) [5, 6]. It may be due to CAC obstructing the delivery and release of stents, resulting in poor stent adhesion, malapposition or direct damage to the stent surface (including polymers), potentially affecting drug delivery [7, 8]. Researchers have found that incomplete stent expansion is the strongest predictor of postoperative stent thrombosis and restenosis [9, 10].

Coronary intravascular lithotripsy (IVL), which combines the principle of transmitting acoustic energy, is a new method to treat atherosclerotic plaque with severe CAC before stent implantation [11]. The safety and effectiveness of IVL in treatment of calcified lesions have been confirmed, and these studies indicate that IVL has a high success rate as an auxiliary method for coronary stent implantation, with good early angiographic and late clinical outcomes [12, 13]. The results of the Disrupt trials indicate that the acute gain of the lumen after IVL use is 1.6 mm2, and the surgical success rate is about 93% [14, 15]. Although these reports provide preliminary evidence and insights into the mechanism of calcium modification, their sample size is relatively small. Additionally, there are few studies comparing the efficacy and safety of IVL and rotational atherectomy (RA) in the treatment of CAC.

The aim of our study is to evaluate clinical outcomes comparing IVL with RA for the treatment of severe CAC in the Chinese population and optimize target lesion preparation for patients with severe CAC.

## Methods

### Study population

A cross-sectional observational trial was conducted in a single center. All subjects were recruited in the Center for Coronary Artery Disease, Beijing An-Zhen Hospital, Capital Medical University. All patients have severe CAC, defined as radiographic opacities on both sides of the arterial wall displayed by fluoroscopy before injection of contrast agent. At the discretion of the operator, the patient receives IVL or RA treatment. Exclusion criteria were as follows: (1) age<18 years old; (2) patients have contraindications or are not suitable for PCI; (3) patients receiving both IVL and RA were excluded. The study protocol was in accordance with the Declaration of Helsinki, and approved by the Ethics Committee of Beijing An-Zhen Hospital of Capital Medical University. All patients signed a written informed consent form to participate in the study prior to any procedures.

### Study device and procedures

The Shockwave Medical (Santa Clara, CA, USA) IVL catheter, which includes two integrated gravel launchers inside the balloon. The diameter range of the IVL catheter is from 2.5 millimeters to 4.0 millimeters, with a length of 12 millimeters. It is connected to the generator through a dedicated connector cable, and the generator is programmed to provide 10 continuous pulses at a speed of 1 pulse/second, with a maximum of 80 pulses per catheter. Select an IVL balloon in a 1:1 ratio of reference vessel diameter, located at the target lesion, inflate to 4 atm, and perform 10 IVL pulses. Then inflate the balloon to 6 atm and repeat this process after 10–15 s [16].

Rotational atherectomy (Boston) consists of a rotating olive shaped burr covered with 2000 to 3000 microscopic diamond chips, which can modify the calcified plaque and change the compliance of blood vessels. Other components include a control console, nitrogen tank, and a turbine started by a foot pedal. Burrs stick to the drive shaft and push through the 0.009-inch Rota-wire. Inject Rota flush solution containing 10,000 units of heparin into the drive shaft (in a 1 L saline bag) to minimize heat and friction between the device and Rota-wire [17].

### Data collection and endpoints

Baseline demographics and clinical characteristics, echocardiography results, procedural details including angiographic and in-hospital outcomes were collected from electronic medical record review and were compared between those who underwent IVL or RA. All patients underwent echocardiography examination. The success of an IVL device is defined as the ability to pass the IVL catheter through the target lesion and transmit lithotripsy pulses. The success of RA is defined as the successful burr passing through the target lesion without burr retention. The effectiveness primary outcome was procedural success, defined as successful stent implantation, residual vascular stenosis less than 30% and no major adverse cardiac events (MACE) in the hospital. In-hospital MACE is defined as all-cause mortality, non-fatal myocardial infarction (MI), or requiring emergency target vessel revascularization (TVR). Safety endpoints include perioperative complications (flow‐limiting dissection, perforation, slow/no reflow, cardiac tamponade).

### Statistical analysis

Categorical variables were reported as frequencies (percentage), while continuous variables were reported as mean ± standard deviation or median and quartile range (25,75 percentiles). The categorical variables were tested using chi square or Fisher’s exact test. Continuous variables were tested for the differences with one-way ANOVA or the Kruskal-Wallis H test. Continuous variables were tested for normal distribution using the Kolmogorov–Smirnov test. A value of *P* < 0.05 was considered statistically significant. All the statistical analyses were performed using IBM-SPSS version 24.0.

## Results

### Baseline characteristics

This study included 152 patients who underwent IVL and 238 patients who underwent RA from January 2023 to November 2023. Baseline characteristics are displayed in Table 1. Regarding demographic characteristics, the gender proportion, medical history of PCI and smoking history among groups reach statistical significance. Unstable angina (UA) was the primary indication for PCI in over 60% of the patients in both groups. In the evaluation of cardiac function, there was no difference in echography between groups.

**Table 1:** Basic clinical characteristics

	IVL (*N* = 152)	RA (*N* = 238)	*P*
Age, years	65 ± 8	66 ± 8	0.685
Male, n,	114 (75.0%)	156 (65.5%)	0.049
BMI, kg/m2	25.23 ± 2.85	25.96 ± 3.33	0.187
Previous MI, n, %	19 (12.5%)	27 (11.3%)	0.730
Previous PCI, n, %	57 (37.5%)	66 (27.7%)	0.043
Previous stroke, n, %	15 (9.9%)	12 (16.5%)	0.067
Hypertension, n, %	98 (64.5%)	164 (68.9%)	0.363
Diabetes mellitus, n, %	63 (41.4%)	114 (47.9%)	0.212
Chronic kidney disease, n, %	6 (3.9%)	6 (2.5%)	0.621
Current smoker, n, %	82 (53.9%)	91(38.2%)	0.002
Acute coronary syndrome			<0.001
UA	104 (68.4%)	156 (65.5%)	
NSTEMI	8 (5.3%)	15 (6.3%)	
STEMI	40 (26.3%)	67 (28.2%)	
Echocardiography			
LVEDD, mm	46 ± 4	46 ± 5	0.227
LVESD, mm	30 ± 4	29 ± 5	0.135
LVEF, %	60 ± 7	61 ± 7	0.316

IVL, intravascular lithotripsy; RA, rotational atherectomy; BMI, body mass index; MI, myocardial infarction; PCI, percutaneous coronary intervention; UA, unstable angina; NSTEMI, non-ST-elevation myocardial infarction; STEMI, ST-elevation myocardial infarction; LVEDD, left ventricular end diastolic diameter; LVESD, left ventricular end systolic diameter; LVEF, left ventricular ejection

### Procedures characteristics

Procedures characteristics are outlined in Table 2. Left anterior descending (LAD) and right coronary artery (RCA) were the main vessels treated in both groups. The 2.5 and 3.0 mm IVL balloons and 1.5 mm burr were the most commonly used. The length of lesions and the total length of stent implantation in the IVL group were smaller than those in the RA group, but the difference was not statistically significant. The use of endovascular imaging was higher in IVL than in RA group (56.2% vs. 40.1%). In our study, radial artery approach was the main approach in both treatment groups.

**Table 2:** Procedural characteristics

	IVL (*N* = 152)	RA (*N* = 238)	*P*
Target vessel			0.446
LM	14 (9.2%)	19 (8.0%)	
LAD	99 (65.1%)	144 (60.8%)	
LCX	5 (3.3%)	16 (6.8%)	
RCA	34(22.4%)	85 (24.5%)	
Largest burr size			
1.25 mm		52 (21.8%)	
1.5 mm		135 (56.7%)	
1.75 mm		48 (20.2%)	
2.0 mm		3 (1.3%)	
IVL balloon diameter			
2.5 mm	57 (37.5%)		
3.0 mm	63 (41.5%)		
3.5 mm	28 (18.4%)		
4.0 mm	4 (2.6%)		
Endovascular imaging			<0.001
Use of OCT, n, %	44 (28.9%)	28 (11.8%)	
Use of IVUS, n, %	40 (26.3%)	65 (27.3%)	
None use, n, %	64 (44.8%)	145 (60.9%)	
Number of stents implanted	1.0 (1.0, 2.0)	2.0 (1.0, 2.0)	0.691
Total stent length, mm	37 (26, 60)	45 (32, 60)	0.055
Mean stent diameter, mm	3.0 (2.75, 3.5)	2.75 (2.5, 3.0)	<0.001
Vascular access			<0.009
Radial artery	150 (98.7%)	221 (92.9%)	
Femoral artery	2 (1.3%)	17 (7.1%)	

IVL, intravascular lithotripsy; RA, rotational atherectomy; LM, left main; LAD, left anterior descending; LCX, left circumflex; RCA, right coronary artery, OCT, optical coherence tomography; IVUS, intravascular ultrasound

### Procedural complications and outcomes

Procedural complications and in-hospital outcomes following IVL and RA treatments are showed in Table 3. During the treatment process, observe the morphological changes of the IVL balloon through X-ray fluoroscopy, and monitor the electrocardiogram and blood pressure changes. 99.3% of cases resulted in successful deployment of DES in the IVL group, which was higher than in the group treated with RA. During hospitalization, there were no serious adverse events (non-fatal myocardial infarction, TVR or death) in the IVL group, but there were two adverse events in the RA group. Procedural complications were higher in the RA group than the IVL group (5.5% vs. 0.7%, *P* = 0.027). There was only 1 case of coronary perforation in IVL group, which was not related with shock wave balloon due to distal guide wire, and the use of small balloon closure did not cause pericardial tamponade.

**Table 3:** Procedural complications and major adverse cardiac events

	IVL (*N* = 152)	RA (*N* = 238)	*P*
Procedural success	151 (99.3%)	228 (95.8%)	0.080
Procedural complications	1 (0.7%)	13 (5.5%)	0.027
Perforation	1 (0.7%)	2 (0.8%)	
Cardiac tamponade	0	1 (0.4%)	
Flow-limiting dissection	0	3 (1.3%)	
Slow/No reflow	0	7 (2.9%)	
MACE	0	2 (0.8%)	*P*>0.9
Non-fatal MI	0	1 (0.4%)	
TVR	0	1 (0.4%)	
All-cause mortality	0	0	

IVL, intravascular lithotripsy; RA, rotational atherectomy; MI, myocardial infarction; TVR, target vessel revascularization; MACE, major adverse cardiac events

## Discussion

This study is the first to evaluate the safety and effectiveness of IVL versus RA for the modification of severe CAC lesions in a Chinese population. The major findings are as follows:

1. IVL was a safe and efficacious tool for CAC plaque modification, with trafficability in most cases.
2. IVL was safe, without reported serious adverse events during hospitalization.

Proper lesion preparation before stent implantation is a prerequisite for successful PCI. However, PCI treatment for severe CAC is challenging as it is associated with an increased risk of perioperative complications (dissection, perforation and slow/no reflow) and inadequate stent expansion, which are predictive factors for stent thrombosis and restenosis [18]. RA has been used in the past to treat severe CAC disease, but according to reports, the hospital mortality rate is 5.4–7.5% [19]. Given such a high incidence rate, it is still crucial to find alternative treatment. The current best alternative therapy for severe CAC is IVL. The results of the Disrupt trials demonstrated the safety and efficacy of coronary IVL treatment for non-LM disease [14, 15]. Our data, though limited in sample size and non‐randomized, supports that patients treated with IVL have less periprocedural complications and in‐hospital MACE.

IVL emits sound pressure waves in a circumferential cross wall manner under low balloon expansion pressure, generating circumferential calcium cracks on multiple planes [20]. Its advantage lies in prioritizing calcium modification without affecting the soft tissue inside the blood vessels. RA burr has only one axis of rotation on Rota-wire, which rotates along the peripheral orbit of the blood vessel to perform plaque ablation. Due to the uniaxial rotation of the burr, it constantly comes into contact with the plaque, producing particles that damage microcirculation function, resulting in slow or no reflow [21]. There was no slow flow/no reflow observed in the use of IVL in the Disrupt studies. Furthermore, the literature reported that perforation rate of approximately 1.7% for RA, while IVL did not observe perforation in previous studies [22, 23]. Another potential advantage of IVL may be in patients with angulated, tortuous and eccentric lesions. Asymmetric CAC may lead to offset of the Rota-wire, which may not guide the burr to the calcified lesion, resulting in insufficient ablation during the advancement process and increasing the risk of burr entrapment. In contrast, IVL could overcome these issues because it does not rely on mechanical tissue damage caused by physical interactions, but rather emits diffuse sound pulses through balloons inflated at low pressures of 4 to 6 atmospheres [13]. IVL or RA are relative contraindications for ST-elevation myocardial infarction (STEMI) patients, who typically have a risk of plaque rupture with thrombosis, but may be the only option for successful surgery for severe calcified culprit lesions. In recent studies on the use of RA or IVL in the setting of STEMI, the success rates of surgery were high and the incidences of MACE were low, which are consistent with this study [24, 25]. Moreover, IVL is more effective than RA in modifying deep calcification, as the impact of shockwave pulses on calcium flakes is independent of their depth in the vascular wall [26]. The advantages of these are that the energy distribution is uniform, which makes the plaque modification uniform, thereby facilitating the expansion and adhesion of the scaffold. Indeed, this different non ablation mechanism may explain lower perioperative complications and in-hospital MACE.

The optimal treatment for severe CAC is multi-adjuvant therapy, which depends on the nature and anatomical distribution of calcified plaques. Given the reassuring results of the Disrupt CAD project, as well as the subsequent multi center registrations in several large real-world settings [27, 28], coupled with the ease of use of the technology, it is expected to quickly be recognized as the preferred treatment for severe CAC before DES implantation. Optical coherence tomography shows that compared to RA, IVL causes more changes in calcified plaques, including longer incisions and deeper faults, which may explain the lower stent misalignment rate and improved stent expansion [26].

The successful use of this device in coronaries for ISR is also very attractive, as calcification often leads to poor stent expansion [29]. In addition, the use of IVL assisted transfemoral catheters in large-diameter catheterization procedure also provide greater possibilities for the application of IVL [30]. However, before selecting a suitable device, consideration must be given to the size limitations of the IVL system (short 12 mm balloon catheter with a diameter range of 2.5–4.0 mm) and the limitations on the number of ultrasound pulses transmitted [16]. Some data indicate that IVL can induce ventricular arrhythmias [31], and it is necessary to continue to pay attention to the safety of this therapy in the future. In summary, in patients with severe CAC requiring revascularization in this study, IVL seems to be feasible compared to RA, with a good initial success rate and incidence of complications.

### Limitation

This study has several limitations. First, this was a small, retrospective study conducted in a single center, with a low evidence-based level. Further validation will be necessary in a larger cohort of patients. Larger scale research is needed to determine the effectiveness and safety of IVL. Second, the endovascular imaging data were not further analyzed in this study. Further analysis of lesion characteristics is needed in the future. Finally, the follow-up time is short and angiographic follow-up were not performed. Cardiac biomarkers were not routinely obtained after PCI.

## Conclusion

This study supports that coronary IVL appears to be safe and effective for the treatment of severe CAC lesions compared to RA.

## Data Availability

The dataset analyzed during the current study are available from the corresponding author on reasonable request.
